# Lectures on the Theory and Practice of Surgery

**Published:** 1845-09

**Authors:** 


### REVIEWS.

---

Art. IV.—*Lectures on the Theory and Practice of Surgery.* By the late Abraham Colles, M. D. For thirty-four years Professor of Surgery in the Royal College of Surgeons in Ireland. Edited by Simon M'Coy, Esq., F. R. C. S. I. Philadelphia: Ed. Barrington & Geo. D. Haswell. 1845. 8vo. pp. 420.

This volume is issued with the July number of the Bulletin of Medical Science, and belongs to the Select Medical Library. The author of it, not now among the living, stood among the foremost in his day for skill and experience, and we are assured that we have in the volume before us not the matter only of his valuable lectures, but the manner also of the eminent teacher.

These lectures begin, as usual, with an account of inflammation, its kinds, characters and causes; but the author does not, as is very common, waste much time in theoretical discussions. His remarks are made with the conciseness of one who had a clear conception of his subject, and a high appreciation of what so many writers under value, time. He imparts instruction in a few words, and hence practitioners will turn with pleasure to his lectures when they wish to refresh their memories upon any practical point in Surgery. On the fourth page he has already reached the treatment of inflammation, and we find him laying down the rules respecting it in the following compendious manner:

"But cold does not agree in every case of local inflammation; sometimes you must apply warmth, and the best criterion—perhaps the only one—by which you are to judge, which is best in any particular instance, is that which the patient feels most agreeable to his sensations. You will be hardly ever wrong when it soothes the part, because this soothing is the very thing we aim at, knowing it is one of our best means to lower the inflammation. Now, warm stupes will often be found to lessen the pain and feeling of tension better than cold, and particularly where the feeling of tension is very strong, and they should be used notwithstanding the objection that they bring more blood to the part; a better objection to them is the slopping and wetting the bed-clothes, teazing an irritable patient and exposing him to cold from their damp. If you use them you will find that in different cases they must be of different degrees of heat; for instance, you can hardly make them hot enough for the comfort of the patient when matter is making its way through tendinous structures. Various medical plants are boiled or macerated in the water of stupes, but I doubt that they are of any use; I am not, however, certain; if any of them do service, it is chamomile, which, I think, I have seen somewhat useful. Poultices are likewise used with nearly the same object as stupes, and, like them, it little matters of what ingredient you make them, provided they are calculated to keep their moisture long. It is absurd to expect a poultice will retain a heat greater than that of the part on which it is put for any length of time; where that is wished for, stuping will be better. Were it not for their weight and the difficulty, in some situations, of keeping them exactly on the part you desire, poultices would be often found very useful, but their only purpose, on a simply inflamed surface, is to keep it soft and moist, and to maintain an equal temperature; if they be applied too hot they will irritate, and continue to do so until they cool down to the natural heat of the skin. Linseed meal worked up in hot water to the proper consistence is very light, and keeps its moisture longer than any other I know of; bread and milk is apt to get dry and hard, and if kept on in this state often does a great deal of harm. Local bleeding is among the means we employ to subdue inflammation; for this purpose scarification, cupping, and leeches are not to be used indiscriminately. To cup and scarify an inflamed part is just adding fuel to fire: leeches alone should be used, and even those put to a highly inflamed part may add to the inflammation already existing, or produce erysipelas. Another objection to leeches is that it is sometimes extremely difficult to stop the bleeding when enough of blood has been taken away, and this notwithstanding everything that you may try for the purpose. This, in an adult, is not of much consequence generally, but I have known more than one child to die of the continuance of the bleeding from leech-bites. You must never forget what a very small quantity of blood an infant can bear to lose without danger, and if a leech or two must be applied to one of them, it should be carefully watched, for the child will fall asleep, and from want of proper caution, may continue to bleed until it is too late to redeem the negligence. Above all things, guard against the use of the Swedish leech, the bite of which will never be obliterated. You should not apply leeches near to each other, for if one bite should ulcerate, it will extend to the neighboring bites, they will run into each other, and sometimes be followed by very unpleasant consequences. On the whole, I do not think leeches by any means so useful as they are represented, except sometimes over inflamed glands, and perhaps in ophthalmia. I need say little of blisters, as no one would be mad enough to put a blister on an inflamed part; after other means have been premised, a blister at some distance will occasionally be very useful, such as to the nape of the neck, or behind the ears, in inflamed eyes. Now, in these cases they do not produce their good effect from the quantity of serum they make to be thrown out, but from counter-irritation, or a kind of sympathy between the two parts. Blisters are serviceable in removing the sequelæ of inflammation, such as the induration that sometimes remains, but on no account are they to be put on, or very near a part in a state of active, acute inflammation."

The second lecture discusses hectic fever, and mortification. Hectic fever is not dependent in every case upon the presence of pus, as we are taught to believe by Cullen and others. Often it is met with where not a particle of pus is to be found in any part of the body. To quote our author—

"You will see, for instance, a woman of delicate constitution fall into decided hectic from suckling her infant longer than the state of her strength or constitution would warrant her in doing, and yet there may not be the slightest indication of any organic derangement to cause the formation of pus, either at the time or in perspective. Here is a case where simple exhaustion brings on the disease, and if the constitution be sound in other respects, its cure is simple and effectual. Strong emotions, or affections of the mind, if continued for any length of time, will, alone, bring on a hectic, which will be fatal if it does not receive proper attention. You see an example of this in the disease termed nostalgia by authors—where a conscript or recruit is forced to leave his family and his country, for the first time it may be, with small hopes of ever seeing either again. In neither of these instances, nor in many others that might be cited, is any pus formed; and that there is no mistake as to the actual cause of the hectic is proved by the fact, that it is effectually cured by making the woman wean her child, and sending the man back to the persons and places for which he yearns. Many cases of hectic present themselves where either no pus is formed at all, or where there is no proportion between the fever and the local affection supposed to be its cause. In hectic from a diseased joint the quantity of pus may be extremely small; and, on the other hand, we have cases where large collections of matter have been absorbed, by means of a successful line of treatment, or some change in the part or the system, and not a sign of hectic will be developed from beginning to end. There does not seem to be anything in the qualities of simple healthy pus that would lead us to think that it ought to cause so serious an effect on the constitution; it is a mild, bland, semi-fluid secretion, unirritating, tardily yielding to putrefaction, sometimes lying quietly encysted, for months, in the body: and for even years continuing to be secreted from the surface of an issue or other ulcer, without prejudice to the parts with which it has been in contact. We are still in the dark as to its uses, but, except from circumstances wholly unconnected with its particular nature, we see no injurious effects from its presence."

The diagnosis of this fever is important, and we copy again from this lecture:

"How are we to know when a patient is hectic? It is often extremely difficult to decide the point in surgical diseases, although easy enough in others, as in phthisis. In surgical hectic the symptoms are never so regular as in others. Hectic fever is often preceded for weeks or months by wandering pains in the limbs like rhumatic pains; here the greatest caution is necessary on the part of the surgeon, for if the patient has had the venereal disease, and that he was treated irregularly, they might be mistaken for venereal pains, and if mercury be, in consequence, exhibited, the patient is driven rapidly into the worst stage of hectic. As soon as the fever sets fairly in, those pains gradually subside. The first symptoms of hectic may be easily, and in fact frequently are, unnoticed; it is so irregular in the occurrence of its paroxysms, that in any six days of its duration there will not be three of these days in which the paroxysm will happen at the same hour. The patient at first only feels a little weakness; he is conscious of not being able to use as much exertion as he used to do, and his friends remark he does not look so well; he rapidly loses flesh; yet his appetite may be as good as usual, and his functions go on regularly. It is soon perceived that once or twice a day he gets a change; about noon a shivering comes on, and lasts perhaps for half an hour, during which he looks pale, and his countenance drawn in; after this the warmth returns and the heat soon increases beyond the standard of comfort, particularly in the palms of his hands, &c., which have a peculiarly biting hot feel. There is nothing in this cold and hot stage of hectic like the corresponding symptoms of most other fevers. He may, without arousing any particular attention in his attendants at the incident, get his chair drawn nearer to the chimney, have the fire stirred, and something wrapped round his feet; by and by, he complains that the fire is too brisk, moves away from it, has a screen interposed, causes the door to be left open, and that's all. Sometimes the chilliness returns during the hot stage, or there comes on the sweating stage (which in hectic is not a general full perspiration over the whole body), and on its subsidence leaves the patient pretty well until the next attack, which usually comes on about six or seven o'clock in the evening. The sweating is seldom perfect; it is generally over the chest and arms, but sometimes on the lower extremities alone; it is not like the sweat in some other cases, but a greasy kind of moisture. The appetite, as I have said, remains tolerably good, and the thirst is less than in other fevers. As it goes on, the patient still loses flesh and strength; the appetite begins to declne; his tongue continues clean, but redder than natural; his mind becomes easily excited; his pulse is about 90, seldom gets as high as 120; his countenance changes; his forehead seems larger, from the falling off of his hair; his eyes get a pearly-white appearance, and sometimes are peculiarly brilliant; he flushes often, or there is a circumscribed redness on one or both of the cheeks; his nose is drawn in, and his finger nails become incurvated. A colliquative diarrhœa generally occurs in the morning, and when this is the case he sinks rapidly; not, indeed, as some imagine, from the consequences of the diarrhœa itself, but from the extent to which the fever has arrived. He will complain of a slight sore throat perhaps, and on examination, a number of white specks called apthæ are seen on the soft palate, or perhaps on the tongue, or under the tongue; or what is more frequently the case, there will be seen a large ulcer in the throat. When these apthæ, or this ulcer appear, you may despair of your patient—he will not recover. Towards the close he is worn to a skeleton, and is weak as an infant. Although the intellect remains little impaired until a short time before death, when there is a slight delirium, his mind partakes of the general weakness; his legs become œdematous; his urine comes away insensibly, and he no longer calls for the bed-pan; he falls into slumberings, and at length dies off exhausted—often so quietly that those about his bed may be awhile unconscious of the event."

Mortification is the result of certain kinds of inflammation rather than of others. It rarely succeeds to rheumatism, but often follows erysipelas. In anthrax inflammation and mortification progress *pari passu.* Concerning the causes of this formidable result the author says:

"The exciting causes of mortification are sometimes extremely slight; for instance, if a patient, laboring under anasarca, gets a scratch of a pin, or a surgeon punctures a part with a fine lancet to let out the fluid, in the great majority of cases this scratch or puncture will end in mortification; in another individual mortification will extend from a common boil. It is a curious fact that when gangrene arises from these slight causes its progress is much slower, and when it is to end fatally the patient lingers longer than when it attacks a robust healthy man."

We have, at present, such a patient under our care. From an abrasion of the scrotum, resulting from the motion inevitable in traveling, sphacelus occurred to a limited extent, but although this took place more than a month ago the old man is still alive, but with an affection of the heart which renders the permanent removal of the dropsical effusion hopeless. At times the pulse of this patient can scarcely be felt at the wrist; his extremities are cold, and he seems more dead than alive; still the mortification of the scrotum did not spread, and the parts show a tendency to heal.

Our author continues on the same subject:

"There are causes independently of any local injury which produce mortification. I have heard it remarked by persons connected with large lunatic asylums in Ireland, that the unfortunate inmates of those institutions are particularly subject to mortification of the feet, without any apparent local circumstance to account for it. Excessive cold of a part will cause mortification of it, but sometimes a much less degree of cold than that of freezing will do it. A man, suppose, gets a fever, which, as it is the fashion, we must call typhus; well, on the eighth or ninth day one of his legs is observed to become colder than the other; it is not merely that the patient himself feels it colder, but there is an actual reduction of its temperature; the next day perhaps it is insensible; sometimes no pain is felt in the limb, and very rarely is there any considerable pain; the leg has sometimes a fulness in it, and sometimes it is shrivelled, and it quickly falls into gangrene. Cases of this kind have occurred where the foot mortified, and dropped off at the ankle, after a slow process. The most we can do generally in such cases is, when we feel one of the bones perfectly loose, to remove it gently with a forceps; or if the gangrene, suppose, stops at the middle of the leg, to wait with patience until the ulcerated surface of the living portion looks clean round the tibia and fibula, by which alone the inferior portion is retained, and then to saw through the bones and liberate it. I have never, with but one exception, known this occurrence to take place in those who were comfortably lodged in a wholesome dry place; it is always among the poorer classes; I have never, in the higher classes, known an individual lose his limb from fever, but my experience of fever among the wealthy is very limited, it being chiefly derived from hospital practice. Well, how are we to treat such a case? not by antiphlogistics certainly; the only thing to be done is to prevent any moisture being put to the limb, and to keep it warm with hot flannels, &c. Suppose you were to go to amputate this limb, you would find it the most troublesome and the nastiest operation that can well be thought of. Sometimes in fever the skin covering the prominences of the scapula, scrotum, hips, trochanters, &c., becomes excoriated, gets red, and becomes mortified in the centre; this arises from pressure and extreme debility, and the most you can do is to apply cushions of curled hair in chamois leather under the hollows of the body to equalize its weight on every part; local applications to the mortified parts are worth nothing, but when they first become red you might apply spirits, either alone or mixed with vinegar, and this will often stop the progress of the local affection. When the cuticle is abraded, lay a thin sheet of gold-beater's leaf on the part; white of egg spread on linen or thin leather will be found useful to prevent these abrasions. After the fever has subsided and the patient is getting well, perhaps getting fat and walking about, still one of those gangrenous spots may show no disposition to heal; should the part be the prominence of the sacrum and the slough be attached to the tendinous aponeurosis covering it, don't be in a hurry to take your instruments to cut it off. If you do, tetanus will in all probability be the consequence; cutting off sloughs in any case, except where they are completely detached from the living parts, and only retained by their continuity with a small portion still connected below, is a foolish and very dangerous practice. I knew a young surgeon, who, getting impatient at the slough of a caustic issue he had made, not separating as soon as he thought it ought, took a scissors and cut it off, and the patient died of tetanus. Gangrene from cold comes on in two ways:—A person, for instance, walking in very cold weather on ice, without shoes or stockings, finds, in the evening, a stiffness in his leg, and without any very apparent pain or inflammation it falls into gangrene. While in another such case a very high degree of inflammation takes possession of the limb before gangrene occurs. In a part affected by excessive cold you must not apply heat suddenly; its vitality has been lowered, and is therefore unable to bear the stimulus of heat in the first instance, as it would cause the death of the part. You must make the transition to its natural temperature very gradually and cautiously. If a part be in a state of a high acute inflammation, we should be equally cautious of applying anything to it that would be likely to reduce its temperature too rapidly, such as ice; the *cold* in this case would likewise be too strong a stimulant for the power in the part to resist its excitement, and the same consequence would follow as the application of heat in the first instance to a frozen part. Your first application, then, perhaps will be snow, the next cold water, then water a little heated, and so on; these should be applied with friction. Notwithstanding our best care, should reaction set in too violently, we have then only to treat the case as we would one of common inflammation."

A modification of gangrene not often met with, we apprehend, by practitioners of this country is thus represented:

"There is another variety of mortification called *cancrum oris* found chiefly in children—very seldom, however, among those of the wealthier classes, but among those feeding on poor, unwholesome diet—and left, perhaps, neglected in their wetted clothes or bed, habitually. So frequent was this disease in the Foundling Hospital, that it was considered a kind of endemical complaint there from year to year. Children that appear particularly subject to exanthematous diseases are not unfrequently affected with this also. The first symptom perceived of it is a fulness in one of the cheeks, and if you put your fingers into the child's mouth, and feel the part from within and without, you will find that the cheek is really thickened; there is no particular redness to be seen in it on the outside, but after some time a red line appears forming around the tumefied part, and sooner or later all within that line will slough out. Sometimes the whole cheek will come away, and the disease often proves fatal. At first all you can do is to poultice the part and sustain the child's strength by suitable nourishing diet—attention to cleanliness, warmth, &c., and the exhibition of bark and other tonics. When the slough separates you are to endeavor to arrest the further progress of the disease by pencilling the edges and surfaces with strong muriatic acid, or, what I prefer, the muriate or butter of antimony."

The third lecture is chiefly taken up with erysipelas, of which so much has been said of late in this Journal, that no one would be likely to read anything that we might copy from the volume before us.

Wounds of various kinds are treated of in the succeeding lecture; namely: Simple, lacerated, punctured, and gun-shot wounds, to which are subjoined some highly valuable remarks on the nervous affections attendant upon such injuries. They will be read with interest, and as it is our design to give our readers the fairest opportunity of judging for themselves of the merits of these lectures we shall make long extracts from this. The veteran teacher says:

"There is a set of symptoms attending gun-shot and other wounds, of which I have as yet said nothing—they are called *nervous;* for instance, a man gets a wound or a severe compound fracture, and on the second or third day, when you visit him, you find him with his eyes blood-shot, he in a high state of delirium, and remember this man was a stout healthy man previous to the accident; well, in this delirium he has his leg dangling perhaps by a bit of tendon, or a little muscle or skin; yet he feels no pain in his limb; he throws and tosses it about as if it did not belong to him; now, what are you to do here? You are to bleed him most largely; until he faints; bleed him instantly, and from such an opening as you would be almost afraid to make in a vein; if the vein you open does not bleed freely, open one in the other arm, and make him sit up; and then, when you find him getting weak, and a cold sweat coming out on his face, you bind up his arm, and most probably he falls into a sound sleep, from which he awakens quite calm and collected. But recollect you must make an impression the first bleeding. You desire an assistant to bleed him, and he tells you he has done pretty well, that he has taken fourteen or sixteen ounces from him; but twenty ounces at least must be taken, or no advantage will be gained. Now, there is another case which you must carefully distinguish from the former; in this case the wound or fracture, or whatever else it may be, has been suppurating for seven or eight days, when on visiting your patient in the morning, you see his eyes suffused; he has a peculiar cast of countenance; he is talking continually; his fingers have a tremor, and so has his tongue, if you bid him put it out. This is a nervous affection, and is peculiar to hard drinkers; nothing could be more injurious than to bleed *this* man—what are we to do? To give him opium—and you must give it in tremendous doses—as large as sixty or seventy drops of tincture of opium every hour, and overpower him with as much as he wishes of whatever he has been accnstomed to drink—if it is whiskey, give him whiskey—if porter, give him porter, and give it in as large quantity and as often as he wishes, until you get him asleep. When a patient in these circumstances gets asleep, he generally recovers; but he may be several days and nights without closing his eyes. A patient was taken into Steevens's Hospital, and he got the first kind of delirium: he was every day getting worse, and we could not account for it; he had a wife who was very fond of him, and she begged hard to be allowed to sit up with him, and so she did—well, the man died, and when he was removed from the bed, two bottles that had contained whiskey were found concealed under the bed; he never had been a hard drinker, but in his delirium he called to his wife for whiskey, which she gave him as often as he called for it, and contrived to elude the vigilance of the nurse, who was a very proper and careful woman. The delirium of the drunkard does not often end badly; but remember you must make inquiries as to what particular intoxicating drink the patient was addicted to, and to administer only that kind during his delirium; for it is a remarkable fact that none other will have the same power as a remedial agent. At our next meeting we will consider some other of these nervous affections arising from local injury."

The subject is continued in the next lecture thus:

"There is another very curious nervous affection arising from these injuries. A man, suppose, in going out of a room, pulls the door after him, and gets his finger jammed in between the door and doorcase. In some days after he feels a kind of creeping sensation going from his finger up his arm, and so to his head, or, as others will say, to their heart; when it arrives there, he is thrown into violent convulsions—these convulsions being of different kinds in different cases, sometimes like epilepsy. Now, it is said by some that these curious affections are owing to a tension of a fascia, but there is no tension in the case I have supposed, for such a case did actually come under my notice. Well—what are we to do here? First see if there is anything in the wound that could cause all this mischief; for sometimes a bit of gravel or a thorn may be the cause of all the disturbance, and which being removed the mischief ceases. I saw a woman who had an abscess in her thigh; it was opened, and the matter let out, but soon she got this affection—indeed the same evening. I came to see her, and on examining the wound, I found the lips glued together, but there seemed a fulness as if there was something which filled the space where the matter of abscess had been. I gently separated the lips of the wound, and there I found a quantity of coagulated blood, which I removed, and she had no farther return of the convulsions. In these cases, if you find nothing suspicious about or in the wound, and if the patient be of a full habit, bleed him, and if that does not cure him, it, at least, prepares the way for the next best remedy—namely, opium, which you must give in large doses. It is not safe to give opium until evacuations have been premised.

"Where you meet a case in which the patient has this sensation of a creeping up the arm, you will find there is nothing that will so speedily put an end to affection as the application of a tourniquet on the limb. A woman fell in James's street; she was a large, stong masculine woman. In falling, her arm came to the ground, and she got a sprain; she was taken into Steevens's Hospital, where a relative of her's was a nurse.

One of the pupils saw her, but did not think much was the matter with her, and supposed she only feigned to be ill. I, however, saw her shortly after, and though there was nothing very serious in the appearance of the injury in her arm, I thought I saw something peculiar in her countenance, and desired her to be kept in till morning. Well, all that night, her shrieks kept every one in the ward awake. The pain went from her thumb up her arm, and as soon as it got to her shoulder, she shrieked violently. It sometimes went from the shoulder down the trunk, and into the thigh of the same side. Every thing that could be thought of was done, but nothing was of the least service. Well, I recollected having read something about the tourniquet in such cases, and so I applied it to her arm, and immediately the next pain came, and advanced as usual, but when it came to the place which was encircled by the tourniquet, it could get no further. I then removed it, and placed it on the thigh, and when the pain came to that part, it was again stopped, and could not get beyond the instrument. I replaced it on the arm, and for several days there was no return of the pain. I then took it off entirely (it was a little cruel, to be sure), but I removed it, however, and quickly all the symptoms returned with the first violence. It was finally replaced on her arm; she had no return of the pain, and was completely cured by this method alone. A woman came up from the country with violent pains in her thighs; they were œdematous, and very much swollen; every remedy had been tried by her medical attendants in the country, but without avail. I was consulted, and I thought it a fair case for the tourniquet. It was applied, and when I went the next day, I heard that she had spent a tranquil night, and slept well, the first night's sleep she had had for some time. Her friends thought me a very great doctor for so expeditiously curing what had so long resisted every one else; but in seven or eight days it lost its efficacy, and ultimately failed. The disease, I believe, was in the spinal marrow. The tourniquet, of course, was not kept always tightened on the limb, for it could not live in that state; but she was instructed to tighten it when she felt the pain coming on."

Tetanus and hydrophobia make the principal part of lectures v. and vi. In respect to the treatment of the former of these affections, the following very judicious observations are made:

"Well, then, as we have no chance of preventing or curing tetanus by any treatment of the wound, we must turn our attention to the constitutional remedies, and what are they? A good deal has been written on the efficacy of opium—with what justice I will not pretend to say. In one case under my own care, which got well, I had ordered two grains of opium every three hours, and when I saw the patient getting better, I thought the opium was doing wonders, but after ten or twelve days I found, on looking at the pills, that they were no larger than the heads of pins—an apothecary's boy who made them up considered a *grain* to mean the smallest possible quantity, and accordingly made them so small that they could have no effect of any kind. If you give opium the tincture is the best form, as the patient can better swallow it; you must begin with forty or fifty drops every two or three hours, and increase the quantity according to the severity of the symptoms, so that in time the dose may be doubled. Even in very large doses opium will not cause drowsiness in a tetanic patient, in one case out of five; where it *does* cause it, it is a favorable sign, and you may then increase the dose; but you may give *too much* opium in this as in other cases, and therefore when you see the patient's eye turgid, as if inflamed, when the face, which was before pale, becomes red and bloated, and its vessels turgid, like an attack of apoplexy, you must lessen the dose immediately; if you persist you will kill your patient as certainly with your remedy as the tetanus would have done. Some say that the bowels become very torpid in this disease—I never saw this, but it may be true, however, and therefore you will look after them, and not suffer them to become too costive, and the best way decidedly to give purgatives in tetanus is in the form of glysters. I recollect when Dr. Hamilton's book came out, it was expected that every disease could be cured by purgatives, and of course they were tried in tetanus; but I always found patients die sooner under this treatment than when nothing of the kind was tried. Musk has been advised; it is, however, too expensive a remedy for hospital use, so I can't speak of it from experience; but from what I have heard of it, I should not be inclined to place much reliance on it. The next best thing to opium is mercury, and the best way to exhibit it is by friction along the spine and elsewhere, in large doses so as to cause salivation as quickly as possible, for there is not much time to be lost; you should throw it in, so as, if possible, to affect the mouth in twenty-four hours. Two drachms of mercurial ointmant should be rubbed in at once along the spine, the limbs, jaws, throat; in fact every where you can, to facilitate its absorption; every inconvenience must be risked if the medicine is to get a fair trial, and this it may get, especially if the case is not a rapid one. Some caution is, however, necessary: you know a patient could be killed by throwing in mercury too quickly. I do not know whether given in tetanus it causus that affection of the heart which it does in other cases, when given incautiously, but I would be on my guard, however. It is difficult to salivate tetanic patients, and we are told that if we succeed in doing so, they will recover; but I have seen patients die of tetanus while under profuse salivation; it has been found more serviceable in the idiopathic than the traumatic species. Warm baths are advised; now, what are their effects here? Just this—the patient may get a paroxysm while being put into it; but the first time he gets into the bath, he feels very comfortable, and wishes to remain in it—the second time he is anxious to get into it, but he does not express so much satisfaction in it as he did the first time; he does not feel quite so comfortable, and you will rarely be able to induce him to try it a third time; the object sought for by the warm bath is to make him sweat. Dr. Wright advises the very opposite of this—namely, the cold bath, or cold water dashed on the patient suddenly, as an infallible cure, but when you come to his cases you find it was not tetanus he was treating at all; for he mentions among others, one case where the paroxysm lasted ten minutes; but in locked-jaw a paroxysm never lasted half that time. I doubt if the doctor ever cured a case by the cold bath; and though he speaks of the disease as it is met with in a warm climate, I doubt his statements, for Dr. Chalmers, a man who certainly wrote from observation, describes the disease in South Carolina exactly as it appears in these countries, and I am convinced there is no difference in its symptoms anywhere. I have heard of patients being cured instantly by the first glass of whiskey on Dr. Rush's stimulant plan, but this was not tetanus, for it was never cured suddenly by any treatment ever employed; but cases of hysteria, bearing some resemblance to tetanus, often are; whenever it has been cured, the improvement has been so very gradual, as to be a character of the disease. A tetanic patient never feels any sensation in the wound that could give him the slightest idea that his sufferings were owing to it."

The editor subjoins the following note detailing a barbarous plan of treating tetanus by the Tonga Islanders. He says:

"I have read in some non-medical work the manner in which the inhabitants of the Tonga Islands cure locked-jaw, to which they are particularly subject from the difficulty of extracting the barbed arrows they employ in battle. They introduce a painted piece of reed into the urethra for three or four inches, and then push the pointed end through its inferior wall and integuments, and leave it there until it causes great inflammation and pain, at which time the rigidity of the muscles passes off, and the person most generally gets well. The late Mr. Wallace had a case in hospital when I was a pupil. I mentioned the savages' cure to him, and he resolved to try it in a modified form. He introduced a bougie smeared with strong red precipitate ointment into the urethra, and as soon as the patient began to suffer pain, the muscles of the limbs grew soft beyond a doubt. The same evening I introduced it myself, the same effect followed, and the man had not another paroxysm for an hour and a quarter, although they had been coming on every twenty minutes previously. The patient, however, actually refused to allow a third application."

The prognosis of tetanus is apt to embarrass the young practitioner. Our author speaks thus in relation to it:

"When cases of common tetanus are to become fatal, we do not find the paroxysm grow less frequent, but they become apparently milder, and, on the contrary, when the patient is recovering, the intervals between the paroxysms are lengthened, but the violence of the last paroxysm that the man may have may be as great as that of any of the preceeding ones. Sometimes on visiting a patient he tells you he is better—he is now able to put two fingers between his teeth, whereas a little while ago he was able to put but one; he *feels* himself better—his jaws can be opened more, and his limbs are more flexible—his friends meet you with a smiling countenance, and everything is congratulation—now what are you to expect? Why, that the next paroxysm the patient gets, will carry him off—the very next paroxysm will certainly be the fatal one. You had better, in the progress of your treatment of a tetanic patient, keep a bit of wood, or cork, which I think better, rolled in a small piece of linen between his teeth, to allow food and medicine to be introduced when necessary; you need not fasten it with a string or anything else. It is said that the body of a tetanic patient runs into decomposition sooner than any other; I cannot say how true or false this may be. Tetanus may end fatally before every other part of the body gets rigid, and patients do very often die before the upper and lower extremities, or both of them, have become so."

Ulcers take up a part of two lectures. It is a subject by no means to be slighted by the student of surgery. These ulcers are often exceedingly troublesome, and require very diverse modes of treatment. The author urges the junior pupil to the most diligent examination of the subject, saying that nothing is so interesting to him in all surgery as the treatment of ulcers. He goes on to say:

"He should dress them with the greatest attention, and observe every appearance and change with care, until he becomes so familiar with them, that he can predict, with certainty, on looking on an ulcer, what will happen in its progress. If your patient is walking about with an irritable ulcer, you may try the soothing plan with it; but the soothing treatment will not answer always. I have seen somewhere a mild poultice almost set the patient mad with pain. I at first thought it might be owing to the kind of poultice used, and have, in consequence changed them repeatedly, but found that whatever poultice was put on produced the same effect, which convinces me that it is not the composition of the poultice, but something in the nature of the ulcer which did not admit of the soothing treatment. I think the term irritable ulcer a very vague one, for any ulcer may become irritable, and with almost any appearance. There is only one description of ulcer I have ever met with, that really deserves the name, and which is not described in books; it is this:—It generally comes on the outer side of the ankle, upon or a little above the external malleolus; it is a small ulcer, sometimes not larger than the point of the finger; it never goes entirely through the skin; it has no edges, nor is there any inflammation about it; it discharges good matter, and has nothing very grave looking connected with it; it is excessively painful, however, and you examine it all over in vain, to say why it is so. You really can see nothing to account for it, in its appearance at least. Now, if you have soft, mild applications, such as poultices laid on it, you will set your patient mad with the pain. You must treat it in directly the reverse way. Rub over its surface with a pipe of lunar caustic, and dress it with a solution of the same, under which the pain will cease, and the ulcer heal."

The discussion of the same subject is contained in the following paragraphs:

"We sometimes meet with an ulcer, the edges of which considerably overhang the surface. You think, on looking at it, that you see the extent of it, but on introducing a probe under these edges, you find that the ulcer extends a good way under it. This is an extremely tedious kind of ulcer, and will keep a patient longer in an hospital than any other I know of. I believe the best application to it is the nitric acid.

"Now, are you, without hesitation, to undertake the healing of any one of these ulcers that you may be called to see? You are not—it would sometimes be a very dangerous thing to succeed in. It does not at all depend on the length of time the ulcer has existed; that it has been a length of time there, discharging, is not at all a reason why you should not attempt to heal it; but if the patient has an affection of the lungs, it is not necessary that he should have a violent cough, with great expectoration; but if he has a thickness of his breathing, and that you discover clearly a tendency to organic disease of his lungs, and that you set about healing his ulcer and succeed, as you may do, you will be, as my old master used to say, his executioner. Don't meddle with it, or you will accelerate his pulmonary complaint, which, with the running ulcer, might have remained long quiescent."

We take the greater pleasure in copying from Dr. Colles's lectures on these minor subjects, because the affections are of more frequent occurrence. They fall out in daily practice, and will meet the young physician at the very threshold of his profession. The management of them makes a part of the regular business of his life, and establishes him in the estimation of the public a skilful or an unskilful practitioner. There is anthrax, for example, and mammary abscess, what physician has not found himself baffled or perplexed by these trivial, but most painful, and often most obstinate affections? How gladly would he have hailed, at such a time, a more successful method of curing them. Respecting the latter of these cases our author has the following:

"I believe active cathartics, of the saline kind, are the very best things you can give. The patient and yourself may be very anxious to have the abscess opened early, on account of the great pain and fever it occasions, but if you do open it you cause much local irritation and profuse night sweats. Leeches do little or no good, and the exposure to cold, the slopping and the irritation they subject the patient to, often does a great deal of harm. Cold applications have been recommended sometimes, but they are not liked by the patient, and I think warm ones are better: your warm fomentations are best applied as women themselves do it—that is, to put the materials of the stupe into a wooden bowl, large enough to contain the breast, and lay it in it, covering the parts outside round with flannel. Even when the abscess feels ripe it is much better not to open it, except indeed the matter be just under the cuticle, when you may just puncture it with a lancet and let out the matter; but in general you ought to avoid the lancet in these cases, for if there be any life or thickness in the part you cut through, you excite fresh inflammation, and greatly increase the chances of other abscesses. [An extremely fetid mass, enough to fill a large whashhand-basin, was here exhibited.] The woman to whom this breast belonged was taken into the hospital for what we all thought was mammary abscess: she, however, died in three weeks, and on examination, we found the mammary gland converted into this firm substance, which is extremely like cow's udder; the fever attending this case, however, was not at all like that of mammary abscess. Whether these abscesses be opened by the surgeon or not, it is ten to one that there will be more than one abscess. Sometimes the inflammation in the breast seems running on to suppuration, and this even for ten days, and after all, matter may not form, but the affection may terminate in resolution."

The eighth lecture is taken up principally with the consideration of injuries of the head, and the subject is pursued through the three succeeding lectures. The author's ample experience in a large city furnished him with the materials for a very instructive series of observations on this important subject. They will be read with interest and advantage, and we should be glad to copy parts of them, but begin to find ourselves already restricted for room.

In the lectures which follow, hare-lip and injuries of the throat are disposed of. We copy the author's description of the operation of tracheotomy:

"When the operation of tracheotomy is to be performed you will find things in a different condition from what you might suppose from performing it on the dead subject. It is all very well to say, throw the head back and give yourself room; but you cannot put your patient into the position you would wish, on account of the difficulty of breathing—it is so great that he cannot bear such a position an instant—and that in which you find him is generally a bad one, for he is leaning forwards; and you must put up with the inconvenience. The way to perform the operation in the upper part of the neck is this:—Take hold of the thyroid cartilage between your finger and thumb, and make a perpendicular incision with a scalpel through the integuments, down to the cricoid cartilage, and then make a transverse cut between the thyroid and cricoid cartilages through the membrane connecting them. Here the parts you operate on are superficial, and no part of consequence in the way of your knife. There is sometimes a little artery running transversely on the crico-thyroid membrane, but even if it should be present, and be wounded, it will give little trouble. A trocar has been recommended to be used in making the opening between the cartilages, but it is a bad iustrument for the purpose, as you might transfix the larynx with it or wound its back part; besides the canula of such an instrument would be a very inconvenient one to leave in the wound, if you thought such a proceeding advisable. I think any canula generally unnecessary in these cases. The moment you put in a canula it is shot from the wound to the other end of the room, and besides, it irritates, although a curved one does so less than a straight one. There is no necessity for a double canula at all, for when you want to clean it, there is no difficulty in removing or replacing it. In operating on the trachea below the thyroid gland in chronic cases, the parts are all so thickened that it is sometimes difficult to find the trachea. I have seen surgeons looking for it before they got between the sternohyoid and thyroid muscles, which will be found in such a case much enlarged in size; a trocar in this latter kind of case is a very dangerous instrument, for it may slip off the trachea, or the trachea may slip from under it, and the instrument be plunged into some of the arteries at the side of the neck. You might make the incision into the trachea with the same knife with which you make the external incision. You should make your division of the integuments long, so as to give you plenty of room, and don't let your assistant draw the lips of the wound asunder, but do it yourself, lest you lose the proper line of your incision, which would embarrass very much the subsequent stages of the operation. You must generally divide some of the veins which lie in front of the trachea, and here you are advised to suspend your proceedings until the blood stops, lest it should get into the trachea, even for two or three hours—but this is not necessary: just get an assistant (and you should take care to provide yourself with an intelligent one for the purpose) and let him draw the lips of the wound asunder with a straight or curved spatula, or put in a dossil of lint on the side the bleeding comes from, and go on with the operation. The trachea lies so loosely connected, that a very slight pressure can move it from side to side, so that before you attempt to open it you must hold it fixed in its place with the finger and thumb of the left hand, while you make your puncture or incision through its rings with the right. You know how closely the carotid artery and jugular vein lie to the side of the trachea, and if your knife slipped off its convexity, it might readily wound one of these vessels: the consequence of such an accident I need not mention. When you have opened the trachea, if you think it necessary, just introduce a quill into the opening, and this will generally be enough. If the operation is required in consequence of a foreign body being in the trachea, it generally happens that the moment this tube is opened the substance that got into it is thrown into the mouth: I don't know how this happens, but so it is."

We have reached, in this superficial examination, the fourteenth lecture, and the volume contains forty-nine; it is therefore evident enough that our notice of the work must be altogether imperfect. Indeed, we did not propose to make anything like an abstract of the volume, but only to extract such passages as would likely be read with interest, and at the same time afford a fair specimen of the matter and manner of the publication. We shall not extend our notice much farther, since the work, from its intrinsic value, and the cheap form in which the publishers have brought it before the profession, is pretty sure of finding its way into the libraries of most of our readers. It strikes us as being a seasonable contribution to our stock of medical literature, a compendious system of surgery having long been a desideratum in this country. We do not undertake to say that these lectures, excellent as they are, fully supply the deficiency; but we have no hesitation in affirming, that they constitute a better body of surgery than is to be found in any volume which has been offered to the American profession in so accessible a form.

A glance at the table of contents will show that all the topics properly belonging to surgery are handled in this volnme: with what fulness and ability the reader may infer from the quotations given. On some points it might be desirable to have more ample details, but in the compass of a little more than four hundred pages it is not possible to say all that it would be profitable to say, on the wide range of subjects embraced in a course of surgery. For this reason we still look for a more complete work—one which, without being as tedious as that of Benjamin Bell, shall embrace all that the student or practitioner ought to know of the present state of surgical science or practice.

In looking over the lectures which remain, we have concluded to make an extract from the one which treats of the diseases of the prostate gland. The author says:

"Among the mechanical obstructions to the flow of urine from the bladder is *enlarged prostate gland:* this enlargement generally, but not always, takes place at the latter periods of life. I saw a man in Edinburgh, not twenty-one years of age, with enlarged prostate, and instances are recorded of its occurrence at a much earlier period. These chronic enlargements are much more frequently met with than attacks of acute inflammation in the prostate, but it must be admitted that the symptoms of the latter are not always of so decided a character, nor so easily distinguished from inflammation of some neighboring parts as to make us quite certain that acute inflammation of the prostate does not happen oftener than is generally supposed. Abscess of the prostate often begins with symptoms closely resembling gonorrhœa; inflammatory fever, more or less well-marked, usually precedes both; there is the same heat and pain in making water; and the pain in micturition is often referred to the same spot in both; there is a discharge from the urethra scarcely purulent perhaps at first, but soon becoming so; but while, in clap, the discharge increases with an uniform progression, in the prostatic disease it will often be observed to be very trifling suppose to-day, profuse to-morrow, again diminished considerably on the next, and so on; even this, however, is not so constant as to be relied on for a distinguishing mark of the nature of the case. There will be often felt a pain or uneasiness in the region of the gland, increased during the passage of hardened stools, irritability of the bladder, or retention of urine. When matter forms in the prostate gland it is sometimes a perfectly good pus, but occasionally it is of that curdy kind usually attributed to a manifestation of a scrofulous constitution. These abscesses open themselves into the neighboring parts. In several of these preparations you observe openings into the urethra from the abscesses in the prostate; in others, they discharge themselves into the rectum, and here is one where the matter would seem to have passed more directly into the cavity of the bladder itself. Sometimes two or three collections of matter form in the gland, either distinct or communicating with each other, and here is a preparation, where, if any of the proper structure of the gland remain, it but forms a thin wall to one large cavity, such as is observed in some cases of collections of matter in the kidney or other glandular organs. Now, here is a preparation where the abscess opens into the urethra, and if you saw the patient when he was alive you would have thought his complaint a very curable one; there was merely a discharge of no great amount from the urethra, which you might call a chronic clap, or gleet, or anything else that came into your head, and yet, as you may well suppose, it baffled everything that was done for him. Here is a preparation of abscess of one of the lateral lobes of the prostate, having two openings by which it discharged its matter—one into the urethra, and the other into the rectum.

Well, all these cases are of course entirely out of our reach."

The treatment, of course, must be varied according to the character and exciting causes of the obstruction. We quote from our author again:

"Home says you can cure the early stage of diseased prostate by preventing the bladder becoming distended with urine, and this you accomplish by not letting it empty *itself*, but using the catheter constantly; but what is the fact? Why, in the first place, you would never get a patient to submit to such a treatment in this stage of the disease, and even if you did, it would be as perfectly useless as every other treatment is that has been from time to time advised. Blisters, leeches, caustics, &c., are quite useless. You might give small doses of calomel and cicuta, as a little benefit has sometimes apparently been derived from them. These diseases of the prostate have nothing malignant in them; true scirrhus of the prostate does sometimes occur, but it is one case in one hundred of enlarged prostate that is true scirrhus, which is one of the most dreadful and terrific diseases that could afflict mankind; the pain is horrible."

Of the surgical treatment we shall not attempt to give any account, but only add, that in a case of enlarged prostate which occurred in our practice some years ago, after the violent symptoms had subsided the patient, a man hardly of middle age, was cured by the use, for several months, of a saline mineral water, by which a gently laxative effect was daily produced upon his bowels.

